# Transumbilical Laparoscopic-Assisted Appendectomy in the Treatment of Acute Uncomplicated Appendicitis in Children

**DOI:** 10.1155/2015/949162

**Published:** 2015-09-29

**Authors:** Carmine Noviello, Mercedes Romano, Ascanio Martino, Giovanni Cobellis

**Affiliations:** Pediatric Surgery Unit, Academic Salesi Children Hospital, Marche Polytechnic University, Ancona, Italy

## Abstract

Transumbilical laparoscopic-assisted appendectomy (TULAA) is increasingly being performed worldwide. The authors report their experience in the treatment of acute uncomplicated appendicitis in children with TULAA. From January 2008 to December 2012 all types of acute appendicitis were divided, according to the clinical and ultrasonographic findings, into complicated (appendiceal mass/abscess, diffuse peritonitis) and uncomplicated. Complicated appendicitis was treated by open appendectomy (OA). All patients with the suspicion of uncomplicated appendicitis were offered TULAA by all surgeons of the team. Conversion to open or laparoscopic appendectomy (LA) was performed in case of impossibility to complete TULAA, depending on the choice of surgeon. The histopathologic examination of appendix was always performed. 444 children (252 males) with acute appendicitis were treated. The mean age was 9.2 years (range, 2 to 14 years). Primary OA was performed in 144 cases. In 300 patients a transumbilical laparoscopic-assisted approach was performed. TULAA was completed in 252 patients. Conversion to OA was performed in 45 patients and to LA in 3. Conversion was related to the impossibility to adequately expose the appendix in 47 patients and bleeding in 1. The mean operative time for TULAA was 42 minutes. Histopathologic examination of the appendix removed by TULAA showed a phlegmonous/gangrenous type in 92.8% of cases. Among the 252 TULAA there were 11 cases of umbilical wound infection. TULAA is a feasible and effective procedure for uncomplicated appendicitis in children. It combines the advantages of open and laparoscopic technique (low operative time, low complications rate, and excellent cosmetic results).

## 1. Introduction

Appendicitis remains the most common emergency surgical condition in children. Since Semm [1] in 1983 described the first laparoscopic appendectomy (LA) using a three-port technique, many institutions performed laparoscopic procedure for pediatric appendicitis. The main benefits of this technique considered are the decreased postoperative pain, the better cosmetic results, and the short hospital stay. In 1992 a laparoscopic appendectomy using a single umbilical puncture was proposed by M. A. Pelosi and M. A. Pelosi III [2] and in 1998, Esposito reported an initial experience in performing one-trocar appendectomy in children [3]. This procedure, named also transumbilical laparoscopic-assisted appendectomy (TULAA) [4], is performed by using only one trocar located in umbilical position and an operative laparoscope: after intra-abdominal mobilization, the appendix is exteriorized through the umbilical incision and resected extracorporeally. TULAA is considered a minimally invasive technique combining simplicity, short operative time, low costs, and low rate of complications [3–10]. TULAA was introduced in our surgical unit in 1997. Later we extended the transumbilical laparoscopic-assisted approach to other techniques as small bowel resection for Meckel's diverticulum and in 2001 we reported our preliminary experience in one-trocar surgery [10].

In this study we present the more recent experience of our centre during a period when all surgeons of the team performed TULAA for the treatment of acute uncomplicated appendicitis.

## 2. Materials and Method

From January 2008 to December 2012 all children referred to our Pediatric Surgery Department for acute uncomplicated appendicitis were operated by TULAA. The diagnosis of appendicitis was based on the clinical presentation (pain to the right lower quadrant, rebound tenderness), fever, blood sample (elevated WBC count and elevated C reactive protein), and ultrasonographic (US) findings [11]. Patients were initially examined by the emergency department physician before the surgical consultation for the suspicion of appendicitis. Until December 2007 TULAA was performed only by surgeons trained in laparoscopic surgery and later all surgeons of the team performed the following approach: every child with clinical and US diagnosis of acute complicated appendicitis (appendiceal abscess/mass, diffuse peritonitis) underwent open appendectomy (OA); patients with acute appendicitis without the suspicion of complication had laparoscopy for TULAA. All patients undergoing surgery were administered Amoxicilline/Acide Clavulanique (50 mg/kg/die in two administration periods) prior and after surgery as long as the inflammatory markers are diminishing. A regimen of Amoxicilline/Acide Clavulanique (50 mg/kg/die) plus Amikacin (7.5 mg/kg/dose twice a day) plus Metronidazole (7.5 mg/kg/dose three times a day) was administered in case of findings of perforated appendicitis.

### 2.1. TULAA Technique

The patient is positioned in the supine position under general anaesthesia. Bladder catheterization is used. A 10 mm ballooned trocar is inserted in “open” fashion through an infraumbilical incision. The pneumoperitoneum is obtained by CO_2_ insufflation (pressure range: 10 to 12 mmHg). Systematic exploration of abdominal cavity is done using a 10 mm operative laparoscope. The appendix is grasped with a laparoscopic atraumatic instrument and pulled through the umbilical incision with the cecum, if mobile. Adhesiolysis is performed in presence of adhesions between appendix, cecum, and peritoneum by bipolar forceps or monopolar hook. Appendectomy is realized outside the abdominal cavity with conventional technique (ligation of the appendiceal vessels, ligation in the basis of the appendix stump, excision of the appendix, and burial of the stump by making a tobacco pouch). The cecum is repositioned inside and a new laparoscopy is performed in order to evaluate the integrity of cecum, bleeding, and presence of eventual content in cavity.

When the appendectomy is considered impossible to be safely completed with transumbilical laparoscopic-assisted approach because of an inadequate exposition and exteriorization through the umbilical incision (some cases of unclear anatomy, inflammatory appendiceal adhesions/mass, retrocecal/subserosal position), a conversion to OA is preferred; as an alternative, depending on the choice of operator, two additional 5 mm trocars are introduced to perform a laparoscopic appendectomy (LA).

Liquid diet can start 12–24 hours after TULAA or later in complicated appendicitis (after canalization). All the appendix stamps removed have a histopathologic examination. All patients had at least one-month follow-up.

## 3. Results

**Figure 1:**
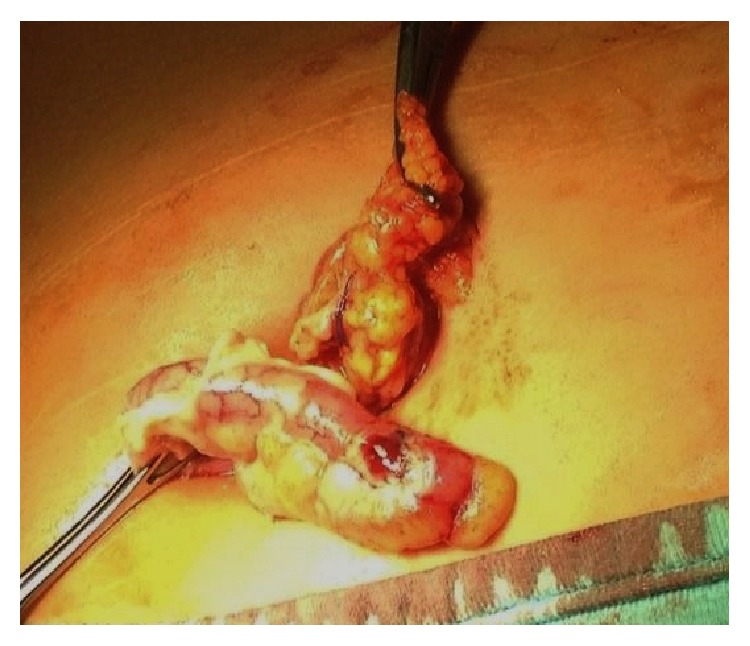
Gangrenous appendicitis exteriorized transumbilically.

During 5 years 444 children (252 males) with diagnosis of acute appendicitis were surgically treated. The mean age at presentation was 9.2 years (range, 2 to 14 years). Primary OA was performed in 144 cases (32.4%) because of suspicion of complicated appendicitis: 123 (85.4%) patients with clinical signs of diffuse peritonitis, 21 (14.6%) with presence of appendiceal mass/abscess (AMA) at US study. Preoperative diagnosis was confirmed at operation in all the cases of AMA and in 86% (106 children) of diffuse peritonitis. An abdominal drain was placed in 41 cases (28.5%). 300 patients with the suspicion of acute uncomplicated appendicitis underwent a transumbilical laparoscopic-assisted approach. TULAA was completed in 252/300 (84.0%). Conversion was necessary in 48 children (16%) (45 OA and 3 LA). The reason of conversion was the impossibility to expose the appendix in 47 cases (98%) and bleeding in 1 case (2%). The histopathologic examination (Table 1) of the appendix stamps removed by TULAA showed a phlegmonous/gangrenous type (Figure 1) in 92.5% of the cases. Moreover a lymphoblastic leukemia was diagnosed in two patients and an* Enterobius vermicularis* was found in the other two cases. Mean operating time to perform TULAA was 42 minutes (range, 20 to 75 minutes) without difference between the surgeons, but depending on the grade of appendicitis and the presence of adhesions. The mean time of antibiotic treatment for patients operated on by TULAA was 4 days (range, 3–5). At follow-up 11 patients (4.4%) operated on by TULAA presented an umbilical wound infection and nobody presented intraperitoneal abscess (Table 2).

Cosmetic results were considered good or excellent by parents in all patients, including those with wound infection. Two children (one with Down syndrome) received a specific treatment for* Enterobius vermicularis* and 2 had haematological treatment for acute lymphoblastic leukemia. The mean hospital stay for TULAA was 4 days (range 3 to 5), and haematological patients were excluded.

## 4. Discussion

Appendicitis is the most common cause of emergency surgery in children. Since the introduction of minimally invasive surgery LA has been widely used and nearly universally accepted for its benefits compared to OA (decreased postoperative pain, better cosmetic results, and short hospital stay). In adults the advantages of laparoscopy seem to be accepted [12, 13], but the same advantages are debated in children. Some authors reported disadvantages of LA versus OA: greater costs, longer operative time, and increased risk for intra-abdominal abscess [14, 15]. Some of these problems are minimized by TULAA. From 1999, when TULAA technique was reported in a large pediatric series [5], several surgeons used this approach for appendicitis in children. The use of only one trocar and reusable instruments can reduce the cost of the laparoscopy [16]. From the technical point of view the small distance between the umbilicus and the cecum in children allows an easier exteriorization of the cecum and appendix [7] through the little umbilical incision compared to adults, with a safe extracorporeal conventional appendectomy and a short operative time. In our opinion in children a technical artifice can be the retraction of umbilical incision towards the cecum to facilitate the appendiceal exposition and resection, even in cases of fixed cecum or retrocecal position of appendix. In the largest pediatric series reported, TULAA was mainly used in cases of acute uncomplicated appendicitis [5, 7]. In a recent retrospective study Codrich et al. [17] reported a series of 181 TULAA (30% of patients had an advanced stage of appendicitis but patients with appendiceal mass/abscess were excluded): they conclude that TULAA is a feasible technique for the whole spectrum of appendicitis, even in cases of perforation. In our series all types of acute uncomplicated appendicitis were treated by transumbilical laparoscopic-assisted approach. We differentiate the surgical approach depending on the clinical and US suspicion of complications. In our opinion TULAA is a feasible technique also in cases of perforated appendicitis on condition that the appendix and the cecum remain adequately movable, despite the inflammatory status and the appendiceal position. However TULAA was not our choice for complicated appendicitis because in these cases it can be more demanding and time consuming, with increased risks of complications (wound infections, abdominal abscess) and conversion rate. Therefore OA or LA is preferred in case of complicated appendicitis. On the other hand the transumbilical laparoscopic-assisted approach can be safely extended to more complex procedures as small bowel resection for Meckel's diverticulum, duplications and hemangiomas, and intestinal biopsies in selected cases [18, 19]. Regarding the feasibility of TULAA in our series it was also performed by surgeons without specific training in laparoscopic surgery and the conversion rate (16%) was not related to the experience of surgeon. In our team the conversion to OA or LA remains a choice of surgeon. Apart from the costs, in children the benefits of one-trocar surgery (low invasivity and cosmesis) are more important than adults, especially in adolescent females, since TULAA can be defined as almost scarless surgery [20]. Moreover in a recent study Montalto et al. founded a significant reduction in the postoperative cytokines in TULAA compared to OA, suggesting a less surgical trauma in children [21]. Finally in our series an histopathologic examination of appendix was always performed and was important for correct diagnosis and postoperative treatment of 2 cases of* Enterobius vermicularis* infection and 2 cases of acute lymphoblastic leukemia.

In conclusion TULAA is a safe and effective procedure with low complication rate for all types of uncomplicated appendicitis in children. This is the optimal procedure because it combines the advantages of open technique (low operative time) and laparoscopic techniques (excellent cosmetic result). In our opinion primary TULAA can be the minimally invasive technique of choice for acute uncomplicated appendicitis in children. Whether TULAA presents advantages over primary OA or LA for complicated appendicitis has to be evaluated in larger prospective studies.

## Figures and Tables

**Table 1:** Histopathologic examination of the all appendix stamps treated (444 children).

	TULAA *n*: 252	OA *n*: 189	LA *n*: 3
Catarrhalis, *n* (%)	11 (4.4)	0	0
Phlegmonous, *n* (%)	155 (61.5)	55 (29.1)	1 (33.3)
Gangrenous, *n* (%)	82 (32.5)	113 (59.8)	2 (66.7)
Perforated, *n* (%)	0	21 (11.1)	0
Appendicular pathology, *n* (%)	4 (1.6)	0	0
Infection by *Enterobius vermicularis*, *n*	2	0	0
Leukemic infiltration, *n*	2	0	0

**Table 2:** Results of the 300 acute uncomplicated appendicitis in patients treated using a transumbilical laparoscopic-assisted approach.

	TULAA	Conversions	
		OA	LA
Patients, *n* (%)	252 (84.0)	45 (15.0)	3 (1.0)
Mean operative time, min (range)	42 (20–75)	50 (40–100)	60 (45–90)
Mean hospital stay, days (range)	4 (3–5)	5 (3–7)	4 (3–5)
Histopathologic examination			
Catarrhalis, *n* (%)	11 (4.4)	0	0
Phlegmonous, *n* (%)	155 (60.3)	36 (80.0)	1 (33.3)
Gangrenous, *n* (%)	82 (32.5)	9 (20.0)	2 (66.7)
Perforated, *n* (%)	0	0	0
Wound infection, *n* (%)	11 (4.4)	4 (8.9)	0
Abdominal abscess, *n* (%)	0	0	0
